# Presenilin Deficiency Increases Susceptibility to Oxidative Damage in Fibroblasts

**DOI:** 10.3389/fnagi.2022.902525

**Published:** 2022-06-16

**Authors:** Kun Zou, Sadequl Islam, Yang Sun, Yuan Gao, Tomohisa Nakamura, Hiroto Komano, Taisuke Tomita, Makoto Michikawa

**Affiliations:** ^1^Department of Biochemistry, Graduate School of Medical Sciences, Nagoya City University, Nagoya, Japan; ^2^Advanced Prevention and Research Laboratory for Dementia, Faculty of Pharmaceutical Sciences, Hokkaido University, Sapporo, Japan; ^3^Laboratory of Neuropathology and Neuroscience, Faculty of Pharmaceutical Sciences, University of Tokyo, Bunkyo City, Japan

**Keywords:** presenilin, oxidative, ferritin, iron, Alzheimer’s disease

## Abstract

Alzheimer’s disease (AD) is a genetic and sporadic neurodegenerative disease characterized by extracellular amyloid-β-protein (Aβ) aggregates as amyloid plaques and neuronal loss in the brain parenchyma of patients. Familial AD (FAD) is found to be genetically linked to missense mutations either in presenilin (PS) or amyloid precursor protein (APP). Most of PS mutations increase Aβ42/Aβ40 ratio, which is thought to result in early amyloid deposition in brain. However, PS deficiency in the fore brain of adult mouse leads to neuronal loss in an Aβ independent manner and the underlying mechanism is largely unknown. In this study, we found that reactive oxygen species (ROS) are increased in PS deficient fibroblasts and that H_2_O_2_ and ferrous sulfate treatment produced more ROS in PS deficient fibroblasts than in wild-type fibroblasts. PS deficient fibroblasts showed significantly decreased cellular ferritin levels compared with wild-type fibroblasts, suggesting reduced iron sequestrating capability in PS deficient cells. Blockade of γ-secretase activity by a γ-secretase inhibitor, DAPT, decreased ferritin levels, indicating that γ-secretase activity is important for maintaining its levels. Moreover, overexpression PS1 mutants in wild-type fibroblasts decreased ferritin light chain levels and enhanced intracellular ROS levels. Our results suggest that dysfunction of PS may reduce intracellular ferritin levels and is involved in AD pathogenesis through increasing susceptibility to oxidative damage.

## Introduction

Alzheimer’s disease (AD) is a neurodegenerative disease characterized by extracellular amyloid plaques in brain parenchyma, intracellular neurofibrillary tangles and neuronal loss (McKhann et al., 1984; Knopman et al., 2021). The major components of amyloid plaques are amyloid β-proteins (Aβ) consisting of 40–43 amino acids, which are generated from amyloid precursor protein (APP) by β-secretase and γ-secretase (Shoji et al., 1992; De Strooper et al., 2012). The accumulation of Aβ occurs 15–20 years prior to AD symptoms and it is one of the earliest pathological events in AD brain (Scheltens et al., 2016). Aβ is hypothesized to be the causative molecule for AD because it has neurotoxic effects, which is called amyloid cascade hypothesis (Selkoe and Hardy, 2016).

AD is clinically divided into sporadic AD (SAD) caused by multiple risk factors, including lifestyle-related factors, and familial AD (FAD) caused by inherited mutations of specific genes. SAD accounts for more than 95% of all AD cases and it is hypothesized to be induced by reduced clearance of Aβ. Genetic causes for FAD include dominantly inherited mutations of the genes encoding amyloid precursor protein (*APP*), presenilin 1 (*PSEN1*, PS1) and *PSEN2* (PS2). PS1 and PS2 provide the catalytic subunit to the γ secretase complex, which contains PS, nicastrin, Aph-1 and PEN-2 (De Strooper et al., 2012). Most of the mutations found in *PSEN* result in increased Aβ42 production or decreased Aβ40 production, leading to an increased Aβ42/Aβ40 ratio (Borchelt et al., 1996; De Strooper et al., 2012; Watanabe and Shen, 2017). The findings of amyloid inhibitory effect of Aβ40 against Aβ42 aggregation and the increased Aβ42/Aβ40 ratio in most of FAD patients with PS mutations provide molecular mechanisms for the pathogenesis of FAD. However, some of *PSEN* mutations lead to reduce Aβ42/Aβ40 ratio and PS conditional knockout mice in the forebrain show progressive neurodegeneration without Aβ deposition, suggesting that loss of PS function could contribute to neuronal damage independently of amyloid cascade (Saura et al., 2004; De Strooper et al., 2012; Watanabe and Shen, 2017).

In addition to amyloid accumulation, oxidative damage in the brain of AD patients also typifies AD pathology and is found to precede Aβ deposition (Nunomura et al., 2001; Ayton et al., 2013; Chen and Zhong, 2014). Redox-active iron is responsible for inducing oxidative damage and extensively deposits in amyloid plaques and neurofibrillary tangles of AD brain (Smith et al., 1997; Zecca et al., 2004; Belaidi and Bush, 2016). We have found that Aβ40 protects neurons against oxidative damage induced by iron, whereas Aβ42 enhanced neurotoxicity (Zou et al., 2002; Zou and Michikawa, 2008). These lines of evidence suggest that Aβ deposition could be a secondary event of oxidative damage in AD brain. Here, we studied whether loss of function of PS is involved in oxidative stress in cells and found that reactive oxygen species (ROS) generation increased in PS deficient fibroblasts compared with wild type fibroblasts. We also found that cellular levels of both ferritin light and heavy chains markedly decreased in PS deficient fibroblasts and that inhibition of γ-secretase activity suppressed cellular levels of ferritin light and heavy chains in the presence of ferrous sulfate. Our results suggest that dysfunction of PS or reduced γ-secretase activity may decrease iron sequestration and enhance oxidative stress.

## Materials and Methods

### Cell Culture and Western Blot Analysis

Wild-type and PS double-knockout (PS-DK) fibroblasts were provided by Dr. Bart De Strooper (Herreman et al., 1999). Cells were cultured in Dulbecco’s modified Eagle medium (DMEM; GIBCO) containing 10% fetal bovine serum (FBS). Wild-type and PS-DK fibroblasts were lysed in RIPA buffer [10 mM Tris/HCl (pH 7.5), 150 mM NaCl, 1% Non-idet P-40, 0.1% sodium dodecyl sulfate (SDS) and 0.2% sodium deoxycholate, containing a protease inhibitor cocktail (Roche)]. Equal amounts (20 μg) of protein from cell lysate were separated by SDS-PAGE in 12% gel and blotted onto polyvinylidene difluoride (PVDF) membranes (Immobilon). The ferritin proteins were detected by Western blotting using rabbit polyclonal anti-ferritin light chain and anti-ferritin heavy chain antibodies (abcam). The blots were visualized using Super Signal Chemiluminescence solution (Wako) according to the manufacturer’s instructions. Membranes were then stripped and re-probed with anti–α-tubulin (Sigma) or anti-β-actin (Proteintec) antibodies. Quantification of protein levels was performed using Image J software (National Institutes of Health).

### Fluorescence Microscopy

The levels of intracellular ROS were measured using dihydroethidium (DHE), which interacts with ROS to form ethidium and emits a red fluorescence in the cells, as described previously (Bindokas et al., 1996). A total of 2 × 10^4^/ml wild-type and PS-DK fibroblasts were seeded and incubated at 37°C in 5% CO_2_ for 12 h and then treated with vehicle control or 1 mM H_2_O_2_. After 30 min, 5 μM DHE (Cayma Chemical) was added into the cells and the cells were incubated for another 30 min at 37°C in 5% CO_2_. Confocal microscopy images were captured using an Olympus FV3000 confocal microscope (Olympus). For quantification of fluorescence intensity, acquired images were imported to Image J software. Threshold intensity was determined using the automatically set thresholding function for all images. Pixel intensity for fluorescence images was expressed as fluorescence mean intensity.

### Reactive Oxygen Species Activity Assay

Intracellular total ROS activity in live cells was analyzed using Amplite Fluorimetric ROS assay kit (AAT Bioquest). WT and PS-DK cells were seeded in a 96 well plate in triplicate and cultured with DMEM containing 10% FBS for 48h. Then the cells were incubated in serum free DMEM and 100 μl/well of Amplite™ ROS Red working solution at 37°C for 1 h. The cells were then treated with 30 and 100 μM FeSO_4_. After 16 h, ROS levels were measured using Amplite Fluorimetric ROS assay kit according to the manufacturer’s protocol. The fluorescence density was measured by plate reader (SPECTRA MAX GEMINI EM) at Ex/Em = 520/605 nm.

### Infection of Presenilin Mutants

The expression of human PS mutants (PS1ΔE9, PS1G384A, PS1D257A, and PS1D385A) was performed as previously described (Islam et al., 2022). Briefly, the retroviral plasmids were transfected into platinum-E cells using FuGENE (Promega) for retroviral packaging. After 24 h, the conditioned medium was collected and used as viral stock. For highly efficient retroviral infection, fibroblasts were cultured with viral stock containing 5 μg/ml polybrene (Santa Cruz Biotechnology) and allowed to proceed for PS expression.

### Statistical Analyses

Prism 7.0 software (GraphPad Software) was used for statistical analyses. All data are shown as the mean ± SEM of at least three independent experiments with *P*-values. *P*-value<0.05 was considered to represent a significant difference. Student’s *t*-test was used to determine whether the results were significantly different between two groups. Group differences were analyzed by one-way analysis of variance (ANOVA) followed by Tukey’s multiple comparison tests for multiple groups against the control group.

## Results

### Reactive Oxygen Species Levels in PS Double-Knockout Fibroblasts Increased at Basal Level and After H_2_O_2_ Treatment

**FIGURE 1:**
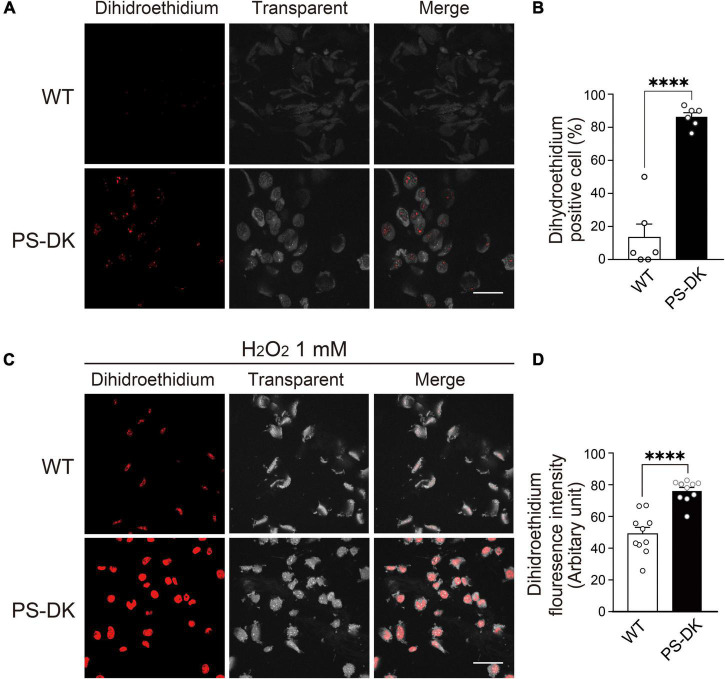
PS deficiency increased oxidative stress in PS-DK fibroblasts. **(A)** Confocal microscopy of DHE staining. Wild-type and PS-DK fibroblasts were incubated with DHE (5 μM) for 30 min in DMEM containing 10% FBS at 37°C in 5% CO_2_. The number of DHE-positive cells increased in PS-DK fibroblasts, indicating that presenilin deficiency enhanced ROS production. **(B)** Ratio of DHE-positive cells in wild-type and PS-DK fibroblasts. *n* = 6 images from 3 independent experiments, ^****^P< 0.0001, unpaired two-tailed Student’s *t*-test. **(C)** Confocal microscopy of DHE staining after H_2_O_2_ treatment. Wild-type and PS-DK fibroblasts were treated with 1 mM H_2_O_2_ for 30 min and then stained with DHE (5 μM). **(D)** Fluorescence intensity in wild-type and PS-DK fibroblasts were determined Image J software. *n* = 10 images from 3 independent experiments, ^****^P< 0.0001, unpaired two-tailed Student’s *t*-test. Scale bars, 20 μm. All quantitative data shown as mean±SEM.

To investigate whether PS function is involved in oxidative stress in cells, wild-type and PS1, 2 double knockout (PS-DK) fibroblasts were stained with DHE, which detects cellular ROS generation by oxidation to form ethidium with red fluorescence. The ratio of DHE-positive PS-DK fibroblasts was significantly higher than wild-type fibroblasts, 13% for wild-type and 86% for PS-DK cells, respectively (Figures 1A,B). This result suggests that the basal ROS levels in PS-DK fibroblasts is higher than wild-type fibroblasts. To examine whether PS-DK fibroblasts are more susceptible to oxidative stress, we treated wild-type and PS-DK fibroblasts with H_2_O_2_ and found that PS-DK fibroblasts generated markedly more ROS than wild-type fibroblasts (Figures 1C,D). This result suggests that PS-DK fibroblasts may have lower antioxidant capabilities than wild-type cells.

### Presenilin Deficiency Leads to Increased Susceptibility to Iron-Induced Oxidative Damage and Decreased Cellular Ferritin Levels

**FIGURE 2:**
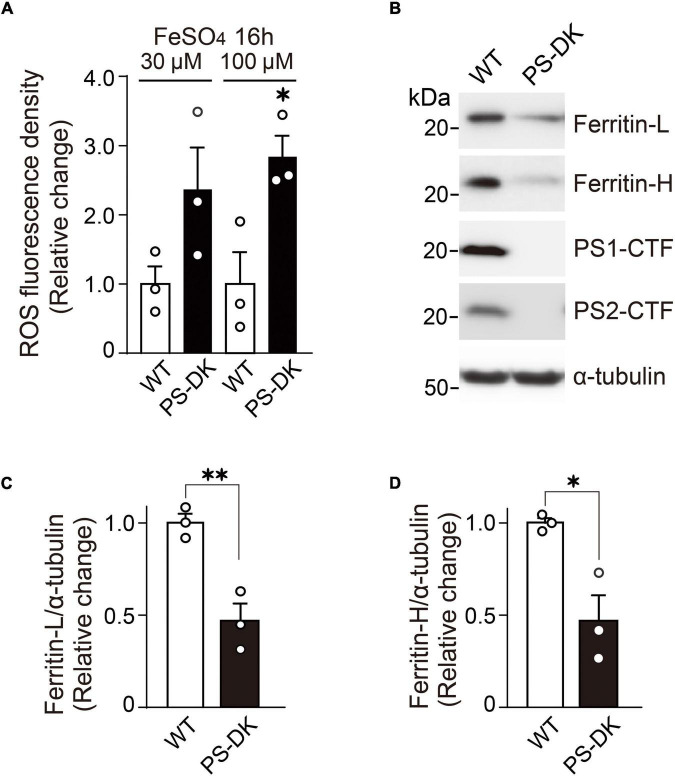
PS-DK fibroblasts generated more ROS by iron-treatment and showed lower intracellular ferritin levels than wild-type fibroblasts. **(A)** ROS fluorescence density were measured 16 h after treatment with ferrous sulfate. PS-DK fibroblasts showed higher ROS levels after iron-treatment. *n* = 3, from 3 independent experiments, *P< 0.05, unpaired two-tailed Student’s *t*-test. **(B)** Western blot analysis of intracellular ferritin light and heavy chains, PS1 C-terminal fragment (CTF) and PS2 CTF. **(C,D)** Quantification of intracellular ferritin light and heavy chain levels. Their levels were normalized to α-tubulin protein levels. *n* = 3, from 3 independent experiments, *P< 0.05, ^**^P< 0.01, unpaired two-tailed Student’s *t*-test. All quantitative data shown as mean±SEM. Ferritin-L, ferritin light chain. Ferritin-H, ferritin heavy chain.

Because H_2_O_2_ generates ROS through Fenton reaction and free iron ion is involved in this reaction, Fe^2+^+H_2_O_2_→Fe^3+^+HO⋅+OH^–^, we examined whether iron treatment can also induce more ROS in PS-DK fibroblasts than in wild-type fibroblasts. The cellular ROS level in PS-DK fibroblasts treated with 30 μM ferrous sulfate showed a tendency toward increase compared with wild-type fibroblasts. The treatment with 100 μM ferrous sulfate significantly increased the cellular ROS level in PS-DK fibroblasts compared with wild-type fibroblasts (Figure 2A). This result suggests that PS-DK fibroblasts have lower antioxidant molecules against iron-induced free radical generation. Antioxidant molecules include direct ROS scavengers and indirect metal binding proteins that quench metal ions to inhibit secondary generation of free radicals. Ferritin is a major intracellular iron binding and storage protein that provides protective effects to cells against free iron-induced oxidative stress (Philpott et al., 2017). We then examined the cellular levels of ferritin in wild-type and PS-DK fibroblasts. Interestingly, total ferritin, ferritin light chain and ferritin heavy chain markedly decreased in PS-DK fibroblasts compared with wild-type fibroblasts (Figures 2B–D). These results suggest that the decrease in ferritin levels may result in increased free iron and increased susceptibility to iron-induced oxidative damage.

### Ferritin Light and Heavy Chains in Response to Iron Treatment Decreased in PS Double-Knockout Fibroblasts

**FIGURE 3:**
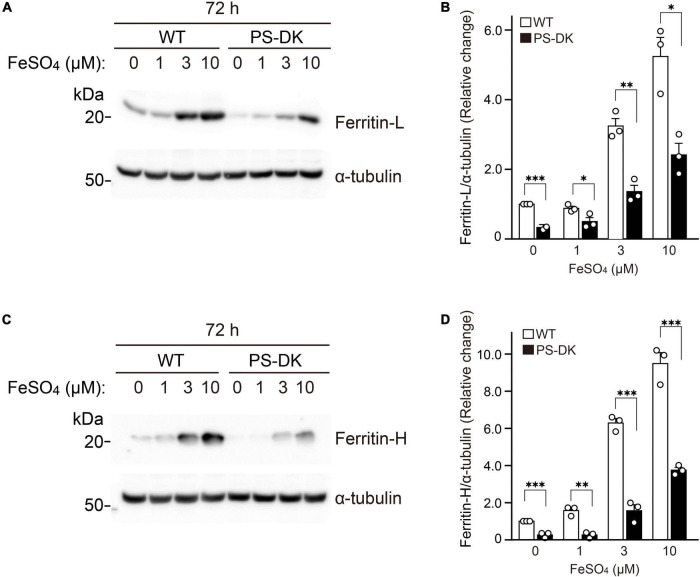
PS deficiency reduced ferritin levels in response to iron treatment**. (A)** Western blot analysis of ferritin light chain in wild-type and PS-DK fibroblasts treated with ferrous sulfate. **(B)** Quantification of intracellular ferritin light chain levels. Their levels were normalized to α-tubulin protein levels. *n* = 3, from 3 independent experiments, *P< 0.05, ^**^P< 0.01, ^***^P< 0.001, one-way ANOVA followed by Tukey’s multiple-comparison tests. **(C)** Western blot analysis of ferritin heavy chain in wild-type and PS-DK fibroblasts treated with ferrous sulfate. **(D)** Quantification of intracellular ferritin heavy chain levels. Their levels were normalized to α-tubulin protein levels. *n* = 3, from 3 independent experiments, ^**^P< 0.01, ^***^P< 0.001, one-way ANOVA followed by Tukey’s multiple-comparison tests. All quantitative data shown as mean ± SEM. Ferritin-L, ferritin light chain. Ferritin-H, ferritin heavy chain.

Ferritin sequesters iron in a non-toxic form and its expression level is regulated by labile iron for protecting cells from damage triggered by excess iron (Picard et al., 1998; Torti and Torti, 2002). We examined whether the regulation of cellular ferritin levels by iron treatment is impaired in PS-DK fibroblasts. Wild-type and PS-DK fibroblasts were treated with 1, 3, and 10 μM ferrous sulfate, respectively. Both wild-type and PS-DK cells showed iron dose-dependent increases in ferritin light chain and ferritin heavy chain levels (Figures 3A,C). However, the cellular levels of ferritin light chain were significantly lower in PS-DK fibroblasts than wild-type fibroblasts at all concentration of iron treatment. Ferritin light chain levels in PS-DK fibroblasts were 57, 42, and 46% compared with wild fibroblasts at 1, 3, and 10 μM ferrous sulfate treatment, respectively (Figure 3B). Ferritin heavy chain levels in PS-DK fibroblasts were 17, 25, and 40% compared with wild fibroblasts at 1, 3, and 10 μM ferrous sulfate treatment, respectively (Figure 3D). These results suggest that PS deficiency does not only reduce the basal level of cellular ferritin, but also reduce the levels of ferritin in response to iron over load. Thus, the increase of ROS levels in PS-DK fibroblasts may result from decreased cellular ferritin levels and increased labile iron ions.

### Inhibition of γ-Secretase Activity Reduced the Increase of Ferritin in Response to Iron Treatment

**FIGURE 4:**
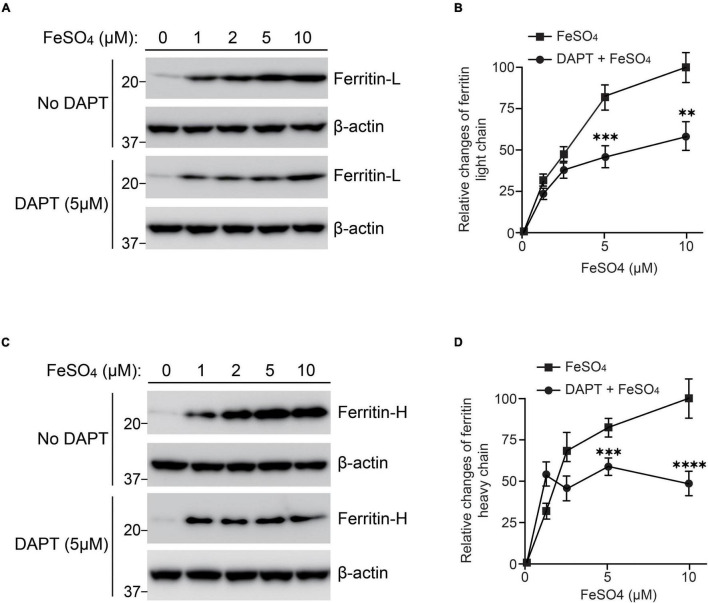
Inhibition of γ-secretase activity reduced ferritin levels in response to iron treatment in wild-type fibroblasts. Wild-type fibroblasts were treated with or without 5 μM DAPT in DMEM containing 10% FBS after seeding. After 1 h, ferrous sulfate was added and the cells were incubated at 37°C in 5% CO_2_ for 72 h. **(A)** Western blot analysis of ferritin light chain in wild-type fibroblasts treated with or without ferrous sulfate or DAPT. **(B)** Quantification of intracellular ferritin light chain levels. Ferritin light chain levels at 10 μM ferrous sulfate treatment (without DAPT) were normalized to α-tubulin protein levels and adjusted to 100%. The relative change of ferritin light chain at each concentration was calculated. DAPT treatment significantly inhibited intracellular ferritin light chain levels. *n* = 3, from 3 independent experiments, ^**^P< 0.01, ^***^P< 0.001, one-way ANOVA followed by Tukey’s multiple-comparison tests. **(C)** Western blot analysis of ferritin heavy chain in wild-type fibroblasts treated with or without ferrous sulfate or DAPT. **(D)** Quantification of intracellular ferritin heavy chain levels. Ferritin heavy chain levels in wild-type fibroblasts with 10 μM ferrous sulfate treatment (without DAPT) were normalized to α-tubulin protein levels and adjusted to 100%. The relative change of ferritin light chain at each concentration was calculated. DAPT treatment significantly inhibited intracellular ferritin heavy chain levels. *n* = 3, from 3 independent experiments, ^***^P< 0.001, ^****^P< 0.0001, one-way ANOVA followed by Tukey’s multiple-comparison tests. ■, ferrous sulfate treatment. 🌑, DAPT and ferrous sulfate treatment. All quantitative data shown as mean±SEM. Ferritin-L, ferritin light chain. Ferritin-H, ferritin heavy chain.

PS is a multifunctional molecule and its major function is serving as a catalytic component of γ-secretase complex (De Strooper et al., 2012). To examine whether γ-secretase activity is involved in regulating ferritin levels in response to iron treatment, wild-type fibroblasts were treated with a γ-secretase inhibitor, DAPT (5 μM), and ferrous sulfate (0, 1, 2, 5, and 10 μM). In the presence of DAPT, ferritin light and heavy chain levels induced by iron treatment was significantly lower than that without DAPT treatment (Figures 4A,C). DAPT treatment suppressed ferritin light chain levels to 37% at 5 μM ferrous sulfate treatment, and to 56% at 10 μM ferrous sulfate treatment compared to those without DAPT treatment (Figure 4B). Ferritin heavy chain levels were also decreased by DAPT to 82% at 5 μM ferrous sulfate treatment and to 53% at 10 μM ferrous sulfate treatment (Figure 4D). These results suggest that γ-secretase activity can regulate ferritin levels in response to iron treatment and that impairment of γ-secretase activity may increase susceptibility to iron-induced oxidative damage.

### Overexpression of Mutant PS1 Decreased Ferritin Light Chain Levels and Increased Reactive Oxygen Species Levels in Wild-Type Fibroblasts

**FIGURE 5:**
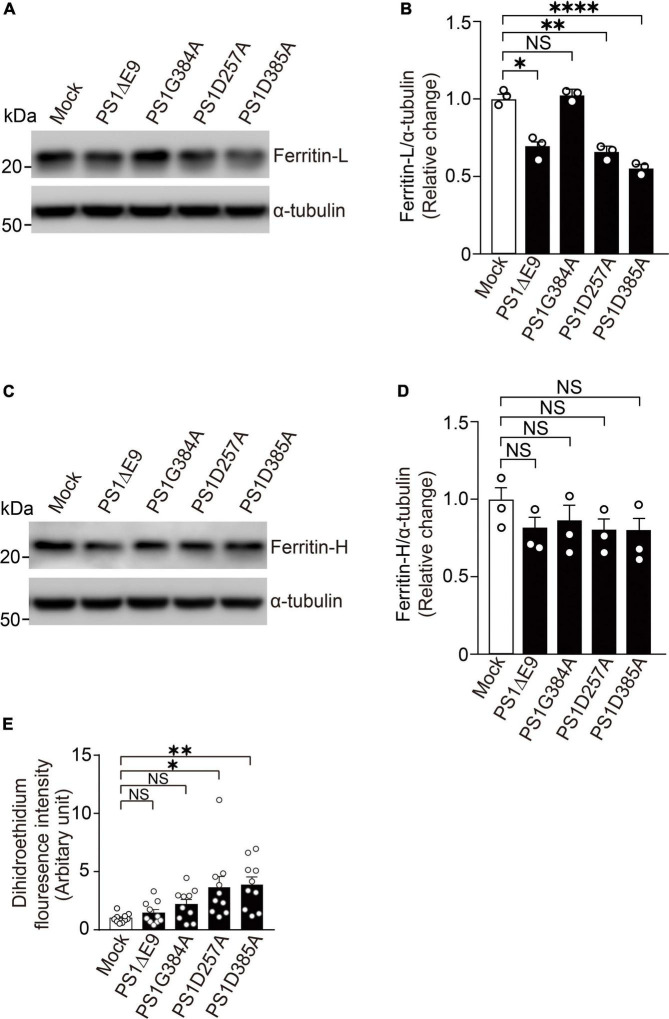
Overexpression of PS1 mutants decreased ferritin light chain levels and increased ROS levels in wild-type fibroblasts. **(A)** Western blot analysis of intracellular ferritin light chain in wild-type fibroblasts overexpressing PS1ΔE9, PS1G384A, PS1D257A, or PS1D385A. **(B)** Quantification of intracellular ferritin light chain levels. Their levels were normalized to α-tubulin protein levels. *n* = 3, from 3 independent experiments, *P< 0.05, ^**^P< 0.01, ^****^P< 0.0001, one-way ANOVA followed by Tukey’s multiple-comparison tests. **(C)** Western blot analysis of intracellular ferritin heavy chain in wild-type fibroblasts overexpressing PS1ΔE9, PS1G384A, PS1D257A, or PS1D385A. **(D)** Quantification of intracellular ferritin heavy chain levels. Their levels were normalized to α-tubulin protein levels. *n* = 3, from 3 independent experiments, NS, not significant, one-way ANOVA followed by Tukey’s multiple-comparison tests. **(E)** Fluorescence intensity in wild-type fibroblasts infected with retrovirus bearing human PS1 mutations (PS1ΔE9, PS1G384A, PS1D257A, and PS1D385A) were determined by Image J software. *n* = 10 images from 3 independent experiments, *P< 0.05, ^**^P< 0.01. NS, not significant, by one-way ANOVA followed by Tukey’s multiple-comparison tests.

To examine whether PS mutants found in FAD (PS1ΔE9 and PS1G384A) or PS mutants without γ-secretase activity (PS1D257A and PS1D385A) can reduce ferritin level and increase ROS level, we overexpressed PS1ΔE9, PS1G384A, PS1D257A, or PS1D385A in wild-type fibroblasts. PS1ΔE9, PS1D257A, and PS1D385A significantly decreased ferritin light chain levels, whereas did not affect ferritin heavy chain levels. PS1G384A did not change ferritin light or heavy chain levels (Figures 5A–D). ROS levels in fibroblasts overexpressing PS1D257A or PS1D385A are markedly more than control cells (Figure 5E). These results suggest that PS mutants with less γ-secretase activity have dominant negative effects on reducing ferritin light chain levels and increasing ROS levels.

## Discussion

Neurodegeneration in the brain of patients with AD has long been considered to be caused by the aggregation of toxic form of Aβ, Aβ42. This notion is strongly supported by the findings that most of genetic mutations in *APP*, *PSEN1*, and *PSEN2* found in FAD showed increased Aβ42 generation or Aβ42/Aβ40 ratio (Scheltens et al., 2016; Sun et al., 2017). However, oxidative damage is found to precede Aβ deposition and iron extensively deposits in amyloid plaques and neurofibrillary tangles in the brain of patient with SAD (Nunomura et al., 2001; Ayton et al., 2013). In support of this oxidative damage pathogenesis theory for AD, we have found that Aβ40, the major form all secreted Aβ, has neuroprotective effects against iron-induced oxidative damage (Zou et al., 2002; Zou and Michikawa, 2008). The findings that iron overload can accelerate Aβ production also support this notion (Becerril-Ortega et al., 2014; Shen et al., 2019). Here we demonstrated that PS deficiency leads to increased ROS generation in cells at basal level and after treatment with H_2_O_2_ or labile iron. In addition, cellular ferritin light and heavy chain levels significantly decreased in PS-DK fibroblasts compared with wild-type fibroblasts, suggesting that the lack of iron sequestration by ferritin increases ROS generation in PS-DK cells.

Multitude of omics studies have characterized expression profiles of thousands of molecular changes and uncovered neuronal gene subnetworks as the most dysregulated in human AD brain and AD mouse models. Interestingly, ferritin heavy and light chain genes, *FTH1* and *FTL*, have been found to be dysregulated in human AD brain and AD mouse models, indicating an important role of ferritin in AD pathophysiology (Bai et al., 2020; Wang et al., 2021). Ferritin plays a key role in maintaining iron homeostasis by capturing and buffering the intracellular labile iron pool (Torti and Torti, 2002). The translation of ferritin is controlled by RNA-binding proteins, iron regulatory protein-1 (IRP1) and IRP2 that interact with iron responsive element (IRE) (Gray and Hentze, 1994; Pantopoulos, 2004; Zhou and Tan, 2017). Microglia highly express FTL with increased Iba1 expression, a marker for activated microglia and represented as iron-accumulating microglia, whereas FTL expressed to a lower extent in neurons in human brain (Kenkhuis et al., 2021). Microglia are in a constant cross-talk with neurons and are capable of tight regulation of neuronal homeostasis. Activated microglia reacts against disease and injury, protect from chronic neuroinflammation and oxidative damage, a hallmark feature of neurodegenerative diseases (Ndayisaba et al., 2019). Thus, PS may be involved in maintaining neuronal homeostasis by regulating iron metabolism in both microglia and neurons.

We found that suppression of γ-secretase activity significantly inhibited the elevation ferritin light and heavy chains in response to iron treatment (Figure 4), suggesting that γ-secretase activity may be involved in IRP- and IRE-mediated pathway in iron metabolism. Among the *PSEN1* mutations examined in this study, PS1ΔE9 causes a reduction in Aβ40 production, whereas PS1G384A mutant significantly increases Aβ42 production, suggesting that PS1ΔE9 results in a decreased γ-secretase activity (Bentahir et al., 2006). PS1D257A and PS1D385A also greatly reduce γ-secretase activity (Wolfe et al., 1999). Thus, the decreased ferritin light chain levels and increase ROS levels in PS1D257A and PS1D385A overexpressing cells may result from loss of γ-secretase activity (Figure 5). It is possible that γ-secretase inhibitors using in clinical trials may reduce ferritin levels or increase ROS levels. This should be studied in human samples from clinical trials in future. There are more than 100 substrates of γ-secretase were identified (De Strooper et al., 2012), that which substrate is responsible for regulating cellular ferritin levels needs to be further studied. Although most of mutations of *PSEN* found in FAD result in impaired γ-secretase activity, dysregulated Aβ42/40 ratio and APP cleavage, our findings suggest FAD *PSEN* mutations may also lead to increased oxidative damage by affecting cellular ferritin levels and provide a novel molecular mechanism underlying the neuronal degeneration in FAD induced by PS mutations.

## Data Availability Statement

The raw data supporting the conclusions of this article will be made available by the authors, without undue reservation.

## Author Contributions

KZ, SI, YS, YG, TN, HK, and TT: data curation. KZ, SI, and YS: data analysis. KZ: conceptualization and writing the manuscript. KZ and MM: supervision and funding. All authors contributed to the article and approved the submitted version.

## Conflict of Interest

The authors declare that the research was conducted in the absence of any commercial or financial relationships that could be construed as a potential conflict of interest.

## Publisher’s Note

All claims expressed in this article are solely those of the authors and do not necessarily represent those of their affiliated organizations, or those of the publisher, the editors and the reviewers. Any product that may be evaluated in this article, or claim that may be made by its manufacturer, is not guaranteed or endorsed by the publisher.
